# New stress-induced hyperglycaemia markers predict prognosis in patients after mechanical thrombectomy

**DOI:** 10.1186/s12883-023-03175-w

**Published:** 2023-03-30

**Authors:** Yi Sun, Yapeng Guo, Yachen Ji, Kangfei Wu, Hao Wang, Lili Yuan, Ke Yang, Qian Yang, Xianjun Huang, Zhiming Zhou

**Affiliations:** Department of Neurology, The First Affiliated Hospital of Wannan Medical College, 2# East Zheshan Road, Wuhu, 241000 People’s Republic of China

**Keywords:** Stress-induced hyperglycaemia, Stress hyperglycaemia ratio, Glycaemic gap, Stroke, Mechanical thrombectomy

## Abstract

**Objective:**

Stress-induced hyperglycaemia (SIH) is a frequent phenomenon that occurs in patients with acute ischaemic stroke. The aim of this study was to investigate the relationship between SIH and the prognosis of mechanical thrombectomy (MT) patients according to the stress hyperglycemia ratio (SHR) and glycaemic gap (GG) indicators, as well as explore its relationship with haemorrhagic transformation (HT).

**Methods:**

Patients were enrolled from January 2019 to September 2021 in our centre. SHR was calculated as fasting blood glucose divided by the A1c-derived average glucose (ADAG). GG was calculated as fasting blood glucose minus ADAG. Logistic regression was used to analyse SHR, GG with outcome and HT.

**Results:**

A total of 423 patients were enrolled in the study. The incidence of SIH was as follows: 191/423 of patients with SHR > 0.89, 169/423 of patients with GG > -0.53. SHR > 0.89 (OR: 2.247, 95% CI: 1.344–3.756, *P* = 0.002) and GG>-0.53 (OR: 2.305, 95% CI: 1.370–3.879, *P* = 0.002) were both associated with poor outcomes (modified Rankin Scale > 2) at Day 90 and an increase risk of HT. Additionlly, receiver operating characteristic curves were used to assess the predictive performance of the SHR and GG on outcomes. The area under the curve for SHR to predict poor outcomes was 0.691, with an optimal cut-off value of 0.89. The area under the curve for GG was 0.682, with an optimal cut-off value of -0.53.

**Conclusion:**

High SHR and high GG are strongly associated with poor 90-day prognosis in MT patients and an increased risk of HT.

**Supplementary Information:**

The online version contains supplementary material available at 10.1186/s12883-023-03175-w.

## Introduction

With the successive publication of several randomized controlled trials, the safety and efficacy of mechanical thrombectomy (MT) have been demonstrated, which has revolutionized the treatment of acute anterior circulation large vessel occlusive stroke [1, 2]. Despite achieving successful recanalization, approximately 50% of patients who are treated with MT fail to achieve a favourable outcome[1]. Moreover admission hyperglycaemia is associated with worse prognosis in patients receiving intravenous thrombolysis [3]. Additionally, it is well acknowledged that hyperglycaemia is strongly associated with poor prognosis and an increased risk of hemorrhagic transformation after MT [3, 4].

Hyperglycaemia is common in ischaemic stroke, not only in diabetic patients with high blood glucose or chronically high blood glucose levels that are undiagnosed, but also in nondiabetic patients with short-term elevated blood glucose levels due to stressful conditions, which we refer to as stress-induced hyperglycaemia (SIH) [5]. SIH is caused by a cascade of reactions resulting from stressors such as trauma and acute illness (stroke), which subsequently cause sympathetic excitation and activation of the pituitary-adrenal axis[5]. Thus, the concentrations of catecholamines, cortisol and inflammatory factors are higher, which leads to a transient increase in glucose, mainly via the process of gluconeogenesis[5, 6].Additionally, the role of insulin resistance (IR) in inflammatory and stressful states should not be ignored [7]. However, some studies have not been able to distinguish well between chronic hyperglycaemic status and stress-induced hyperglycaemia.

There is no uniform definition of SIH. Some studies have defined admission glucose > 200 mg/dl (definitions vary between studies) without diabetes as SIH [8, 9], diabetes diagnosed via previous medical history and/or glycated haemoglobin (HbA1c) > 6.5% [10], which seems to be reasonable, thus ignoring the stressful conditions occurring in diabetic patients. Recently, two new markers have been proposed to assess SIH, which are known as the stress hyperglycaemia ratio (SHR) and glycaemic gap (GG) [11]; these markers, are not based simply on absolute blood glucose levels, but rather on relative values derived from background blood glucose as a reference. SHR and GG markers have been validated in the prognosis of patients with intravenous thrombolysis [12].

Therefore, the purpose of this study was to examine the incidence of SHR and GG in patients undergoing MT and to explore the impact of SIH assessed by SHR and GG on their outcomes.

## Methods

### Study participants

**Fig. 1:**
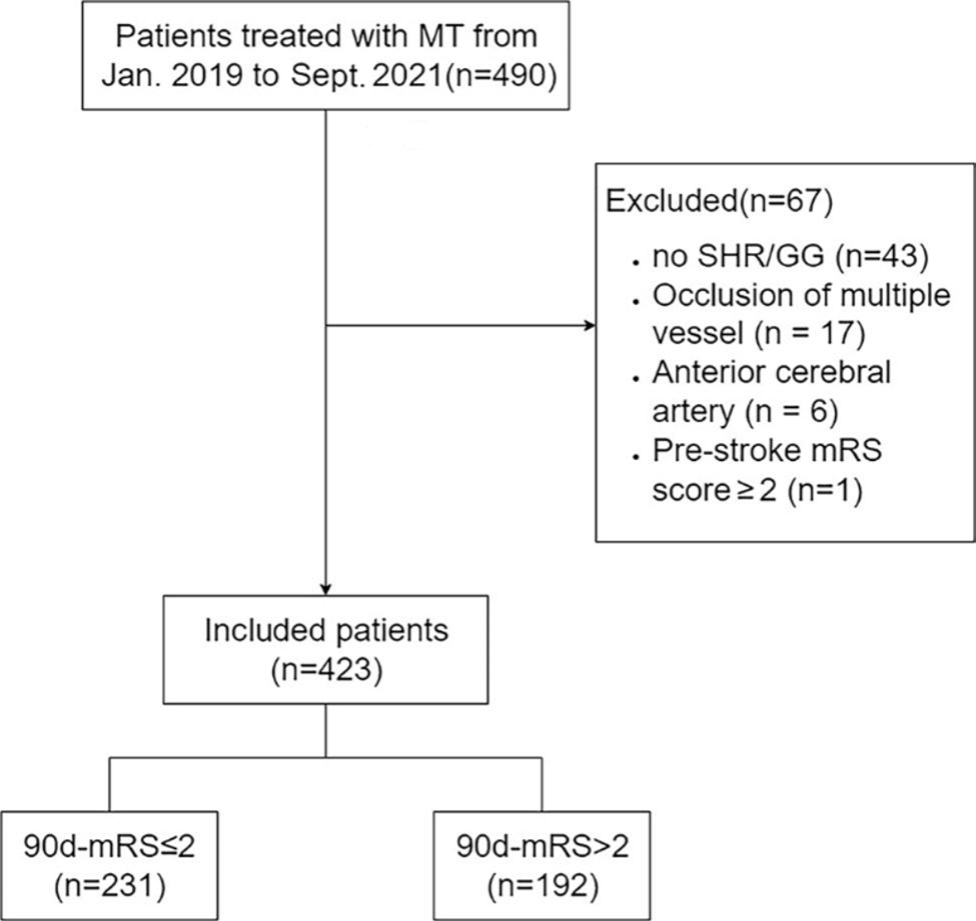
Flow chart of the inclusion of the study population

We continuously enrolled patients with anterior circulation large vessel occlusive stroke who underwent MT at the First Affiliated Hospital of Wannan Medical College from January 2019 to September 2021. The ethics of the study were reviewed and approved by the Ethics Committee of Wannan Medical College. Due to its retrospective nature, informed consent of the patients was waived. The exclusion criteria were as follows: (1) age < 18 years; (2) prestroke modified Rankin Scale (mRS) score ≥ 2; (3) CTA or DSA confirmed occlusion of the anterior cerebral artery, occlusion farther than the M2 segment of the middle cerebral artery, or multivessel occlusion; (4) absence of postoperative imaging data; (5) no postoperative laboratory indices of fasting blood glucose and glycated haemoglobin levels; and (6) no postoperative 90-day mRS score assessed by clinical follow-ups or hospital visit records(Fig. 1).

For all of the included patients, we collected demographic data (age and sex), personal disease history (hypertension, atrial fibrillation, diabetes and antiplatelet/anticoagulant history), clinical data (IT, baseline blood pressure, Trial of Org 10,172 in Acute Stroke Treatment [TOAST] classification, admission National Institutes of Health Stroke Scale [NIHSS] score and admission Alberta Stroke Program Early CT [ASPECT] score), and laboratory data within 24 hours (fasting blood glucose, FBG and glycated hemoglobin, among other parameters.). Assessments and recordings of relevant surgical data by surgical participants included symptom onset-to-puncture time (OTP), onset-to-reperfusion time (OTR), site of occluded vessel, degree of recanalization and collateral status.

### Variable definitions

The condition of the collateral status was assessed according to the extent of contrast reversal within the occluded arterial basin at delayed DSA angiography, with the following parameters being defined: grade 0 was defined as little or no significant contrast reversal within the confines of the occluded vessel; grade 1 was assigned if the collateralization reached the middle cerebral artery M3 segment, and grade 2 was defined if the collaterals reached the middle cerebral artery M2 segment or the distal main stem[13]. We defined a thrombolysis in cerebral infarction (mTICI) score of 2b or 3 as successful recanalization [14]. Functional prognosis was assessed via the 90-day mRS, which we defined as good prognosis for 0–2 and poor prognosis for 3–6.

A1c-derived average glucose(ADAG) is an indicator derived from glycated haemoglobin to assess blood glucose levels over an 8–12 week period [15], and it is calculated as 1.59*HbA1c-2.59.Moreover, SHR is calculated by dividing FBG by ADAG, and GG is calculated by FBG minus ADAG [11].

### Statistical analysis

All of the patients were grouped into favourable and poor outcome groups according to the 90-day mRS scores. Normally distributed continuous variables are expressed as the mean ± standard deviation, nonnormally distributed continuous variables are expressed as quartiles and medians and categorical variables are expressed as percentages/frequencies. For comparisons between the groups (if not specified), chi-square and Fisher tests were used for the categorical variables, t tests were used for normal variables, and nonparametric tests for nonnormal variables. Variables were assessed for normality by using the Kolmogorov-Smirnov test.

Univariate regression analysis was used to assess the relationship between prognosis and clinical characteristics, and variables with *P* < 0.05 in the univariate analysis were included in the regression models. Receiver operating characteristic (ROC) curves were used to evaluate SHR/GG to predict outcomes. Furthermore, the optimal test cut-off point was determined by calculating Youden’s index.

Statistical significance was set at P < 0.05 (two-tailed). Statistical analyses were performed by using SPSS 26.0 (IBM, Armonk, NY, USA).

## Results

### Baseline characteristics

A total of 490 patients with LVOS who received MT were enrolled in this study, and 67 patients were excluded based on the exclusion criteria, with 423 patients ultimately meeting the study criteria.

The median age of all of the examined patients was 70 years, and 250 (59.1%) were male. Of all of the patients, 269 (63.6%) had a history of hypertension, 59 (13.9%) had a history of diabetes mellitus, and 221 (52.2%) had a history of atrial fibrillation. At admission, the median NIHSS and ASPECT scores were 14 (11,17) and 9 (7,10), respectively. The median SHR was 0.87 (0.76, 1.05), and the median GG was − 0.84 (-1.67,0.33). Moreover, 191 (45.2%) of all of the patients had an SHR > 0.89, and 169 (40.0%) patients had a GG > -0.53. In the study population, 379 (89.6%) patients achieved recanalization with grade 2b/3 mTICI (Table 1).

**Table 1:** Univariate analysis of 90⁃day functional independence (mRS ≤ 2) in mechanical thrombectomy patients

	All patients (n = 423)	Good outcomes (n = 231)	Poor outcomes (n = 192)	*P*-value
[year, *M* (*Q*_*1*_, *Q*_*3*_*)*]	70(62,77)	68(58,75)	74(66,78)	< 0.001
Male [n, (%)]	250(59.1)	154(66.7)	96(50.0)	0.001
Medical history [n, (%)]				
Hypertension	269(63.6)	134(58.0)	135(70.3)	0.009
Diabetes mellitus	59(13.9)	26(11.3)	33(17.2)	0.080
Atrial fibrillation	221(52.2)	97(42.0)	124(64.6)	< 0.001
Antiplatelets/anticoagulants history [n, (%)]				0.523
No	340(80.4)	188(81.4)	152(79.2)	
Antiplatelets	58(13.7)	28(12.1)	30(15.6)	
Anticoagulants	25(5.9)	15(6.5)	10(5.2)	
IT [n, (%)]	53(12.1)	28(12.1)	23(12.0)	0.964
Baseline SBP^a^[mmHg, *M* (*Q*_*1*_, *Q*_*3*_*)*]	152(139,168)	150(138,165)	156(140,172)	0.033
Baseline DBP^b^[mmHg, *M* (*Q*_*1*_, *Q*_*3*_*)*]	83(74,92)	83(74,92)	82(74,93)	0.708
Admission NIHSS [*M* (*Q*_*1*_, *Q*_*3*_*)*]	14(11,17)	12(10,15)	15(12,19)	< 0.001
Admission ASPECT [*M* (*Q*_*1*_, *Q*_*3*_*)*]	9(7,10)	9(8,10)	8(6,9)	< 0.001
TOAST classification [n, (%)]				< 0.001
LAA	119(28.1)	87(37.7)	32(16.7)	
Cardioembolic	257(60.8)	116(50.2)	141(73.4)	
Others	47(11.1)	28(12.1)	19(9.9)	
Occlusion location [n, (%)]				0.036
ICA	164(38.8)	78(33.8)	86(44.8)	
MCA(M1)	216(51.1)	131(56.7)	85(44.3)	
MCA(M2)	43(10.2)	22(9.5)	21(10.9)	
OTP [min, *M* (*Q*_*1*_, *Q*_*3*_*)*]	300(230,390)	300(221,360)	300(240,392)	0.424
OTR^c^ [min, *M* (*Q*_*1*_, *Q*_*3*_*)*]	356(280,456)	350(270,454)	360(300,493)	0.105
Collateral score^d^ [n, (%)]				< 0.001
Grade 0	49(11.6)	5(2.2)	44(22.9)	
Grade 1	101(24.0)	43(18.8)	58(30.2)	
Grade 2	271(64.4)	181(79.0)	90(46.9)	
mTICI,2b/3 [n, (%)]	379(89.6)	216(93.5)	163(84.9)	0.004
SHR [*M* (*Q*_*1*_, *Q*_*3*_*)*]	0.87(0.76,1.05)	0.83(0.72,0.94)	0.97(0.83,1.13)	< 0.001
GG [*M* (*Q*_*1*_, *Q*_*3*_*)*]	− 0.84 (-1.67,0.33)	-1.81(-1.07,-0.50)	-1.26 (-0.17,0.86)	< 0.001
SHR (> 0.89) [n, (%)]	191(45.2)	69(29.9)	122(63.5)	< 0.001
GG (>-0.53) [n, (%)]	169(40.0)	59(25.5)	110(57.3)	< 0.001

Abbreviations: mRS, modified Rankin Scale score; SBP, systolic blood pressure; DBP, diastolic blood pressure; 1mmHg = 0.133 kPa; IT, Intravenous Thrombolysis; NIHSS, National Institutes of Health Stroke Scale; ASPECT, Alberta Stroke Program Early CT; TOAST, Trial of Org 10172 in Acute Stroke Treatment; LAA, large-artery atherosclerosis; ICA, internal carotid artery; MCA(M1/M2) M1/M2 middle cerebral artery segment, OTP, onset-to-puncture time; OTR, onset-to-reperfusion time; mTICI, modified Thrombolysis in Cerebral Infarction; SHR, stress hyperglycaemia ratio; GG, glycaemic gap

a:13 patients lost data on SBP

b:13 patients lost data on DBP

c:1 patient lost data on OTR

d:2 patients lost data on Collateral score

### Relationship between SHR, GG and outcome

All of the patients in this study were divided into two groups based on 90-d mRS scores: 231(54.6%) were classified in the favourable outcome group, and the other 192 (45.4%) were classified in the poor outcome group. In the univariate analysis, the percentage of the population with SHR > 0.89 significantly differed between the favourable and poor outcome groups (29.9% vs. 63.5%, *P* < 0.001), and so did GG>-0.53(25.5% vs.57.3%, *P* < 0.001). In addition, compared with the good prognosis group, patients in the poor prognosis group were older (*P* < 0.001), and had a higher proportion of male patients (*P* = 0.001), previous hypertensive disease (*P* = 0.009), embolism (*P* < 0.001) and ICA occlusion(*P* < 0.036);however, they had a lower proportion of good collateral circulation (*P* < 0.001) and successful reperfusion (*P* = 0.004), as well as, a higher median admission NIHSS (*P* < 0.001), a higher median baseline systolic blood pressure (*P* = 0.033), and a lower median admission ASPECT (*P* < 0.001).

**Fig. 2:**
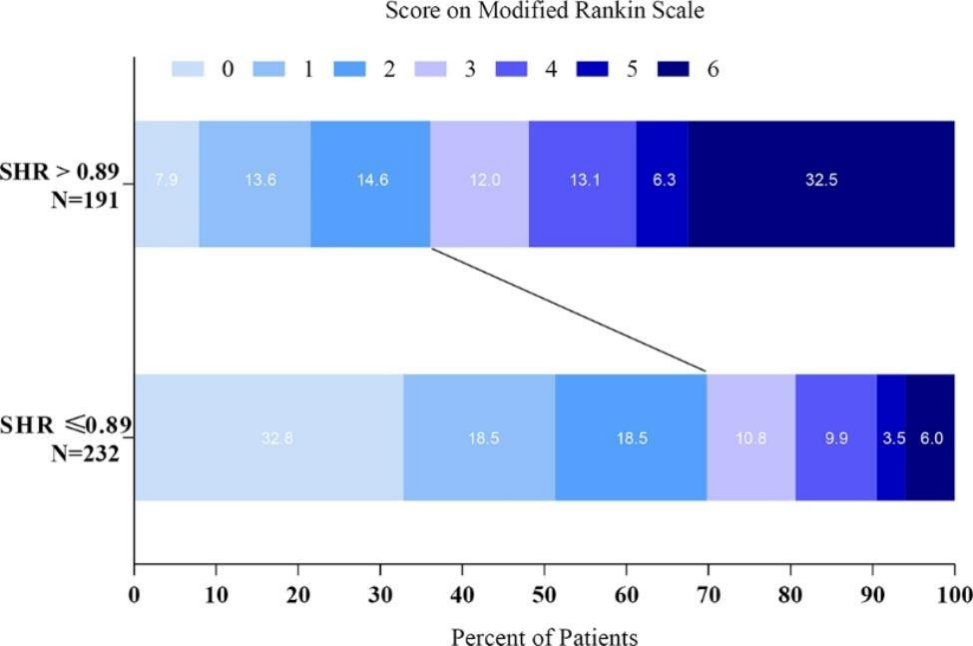
Distribution of modified Rankin Scale (mRS) score at Day 90, according to SHR

**Fig. 3:**
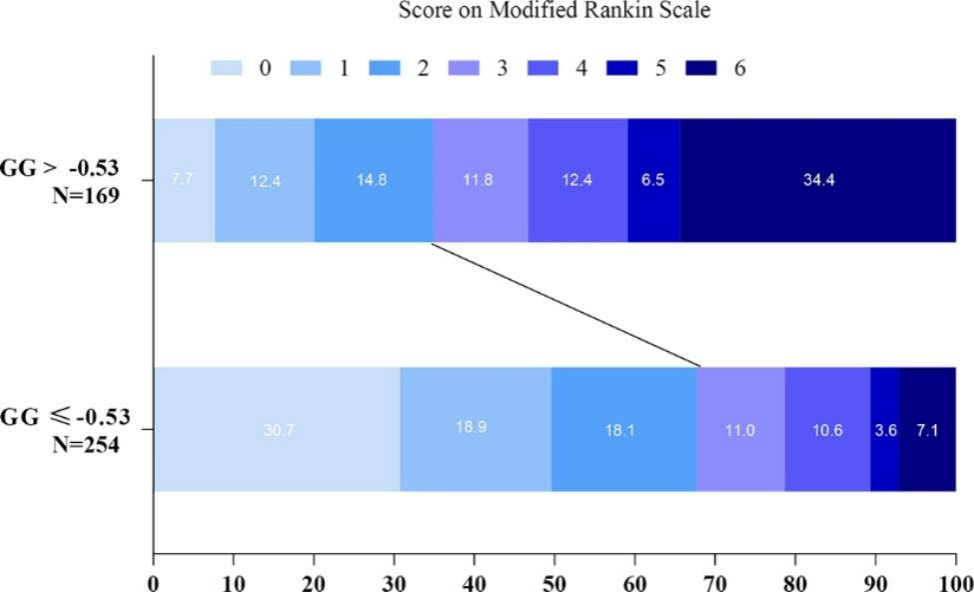
Distribution of modified Rankin Scale (mRS) score at Day 90, according to GG

In both regression models, after adjusting for variables with *P* < 0.05 in the univariate analysis, Model 1 for, SHR > 0.89(OR: 2.247, 95%CI: 1.344–3.756, *P* = 0.002) and Model 2 for GG>-0.53 (OR: 2.305, 95%CI: 1.370–3.879, *P* = 0.002) demonstrated reduced 90-day good functional outcomes (Table 2). The distribution of modified Rankin Scale (mRS) scores at Day 90 according to the SHR and GG groups is shown in (Figs. 2 and 3**)**, respectively. In addition, in Model 1, male (OR: 1.731, 95%CI: 1.029–2.912, *P* = 0.039), high baseline NIHSS (OR: 1.118, 95%CI: 1.050–1.190, *P* < 0.001), low baseline ASPECT (OR: 0.706, 95%CI: 0.604–0.825, *P* < 0.001), and poor collateral score (Grade 1 vs. Grade 0, OR: 0.187, 95%CI: 0.054–0.650, *P* = 0.008; Grade 2 vs. Grade 0, OR: 0.119, 95%CI: 0.036–0.396, *P* = 0.001) were independently associated with poor functional outcomes at 90 days, and in Model 2, male (OR: 1.767, 95%CI: 1.050–2.972, *P* = 0.032), high baseline NIHSS (OR: 1.122, 95%CI: 1.054–1.194, *P* < 0.001), low baseline ASPECT (OR: 0.707,95%CI: 0.604–0.826, *P* < 0.001), and poor collateral circulation (Grade 1 vs. Grade 0, OR: 0.190, 95%CI: 0.055–0.655, *P* = 0.009; Grade 2 vs. Grade 0, OR: 0.120,95%CI: 0.037–0.399, *P* = 0.001)were similar.

**Table 2:** Multivariate analysis of 90⁃day functional independence (mRS ≤ 2) in mechanical thrombectomy patients in Model 1 and Model 2

	Model 1			Model 2		
	Odds Ratio	95%CI	*P*-value	Odds Ratio	95%CI	*P*-value
Age	1.023	0.995 ~ 1.051	0.110	1.024	0.996 ~ 1.052	0.093
Male	1.731	1.029 ~ 2.912	0.039	1.767	1.050 ~ 2.972	0.032
Hypertension	1.324	0.771 ~ 2.274	0.309	1.316	0.766 ~ 2.261	0.319
TOAST classification			0.351			0.388
Cardioembolic vs. LAA	1.474	0.764 ~ 2.842	0.247	1.390	0.717 ~ 2.695	0.329
Others vs. LAA	1.794	0.715 ~ 4.500	0.213	1.814	0.728 ~ 4.518	0.201
Admission NIHSS	1.118	1.050 ~ 1.190	< 0.001	1.122	1.054 ~ 1.194	< 0.001
Admission ASPECT	0.706	0.604 ~ 0.825	< 0.001	0.707	0.604 ~ 0.826	< 0.001
Baseline SBP^a^	1.011	1.000 ~ 1.023	0.060	1.011	0.999 ~ 1.022	0.073
Occlusion location			0.366			0.380
M1 vs. ICA	0.718	0.420 ~ 1.226	0.225	0.714	0.418 ~ 1.220	0.218
M2 vs. ICA	0.610	0.254 ~ 1.463	0.268	0.631	0.263 ~ 1.511	0.301
mTICI,2b/3	0.716	0.303 ~ 1.694	0.446	0.742	0.311 ~ 1.768	0.501
Collateral score^b^			< 0.001			0.001
Grade 1vs Grade 0	0.187	0.054 ~ 0.650	0.008	0.190	0.055 ~ 0.655	0.009
Grade 2vs Grade 0	0.119	0.036 ~ 0.396	0.001	0.121	0.037 ~ 0.399	0.001
SHR (> 0.89)	2.247	1.344 ~ 3.756	0.002			
GG (>-0.53)				2.305	1.370 ~ 3.879	0.002

mRS, modified Rankin Scale score; SBP, systolic blood pressure; DBP, diastolic blood pressure; 1mmHg = 0.133 kPa; NIHSS, National Institutes of Health Stroke Scale; ASPECT, Alberta Stroke Program Early CT; TOAST, Trial of Org 10172 in Acute Stroke Treatment; LAA, large-artery atherosclerosis; ICA, internal carotid artery; MCA(M1/M2) M1/M2 middle cerebral artery segment, mTICI, modified Thrombolysis in Cerebral Infarction; ADAG, A1c-Derived Average Glucose; SHR, stress hyperglycemia ratio; GG, glycemic gap

a:13 patients lost data on SBP.

b:2 patients lost data on Collateral score.

### SHR and GG for Predicting the functional outcomes

**Fig. 4:**
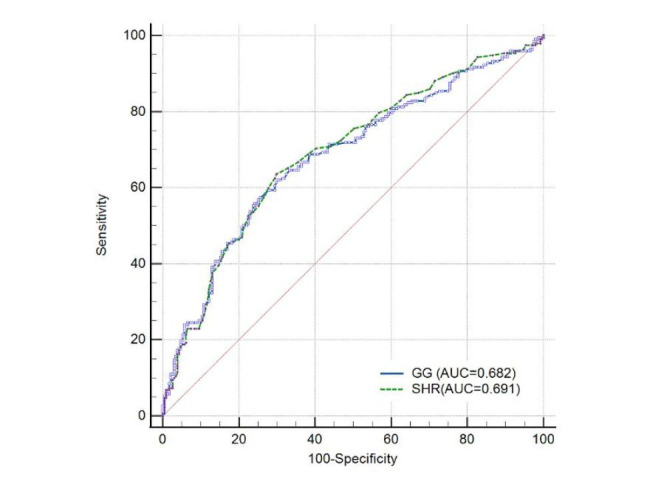
Receiver operating characteristic (ROC) curves showed predictive SHR and GG for functional outcomes. MT, mechanical thrombectomy; mRS, modified Rankin Scale score; SHR, stress hyperglycemia ratio; GG, glycemic gap

Receiver operating characteristic (ROC) curve analysis was used to determine the functional outcomes of SHR and GG for LVOS patients who received MT. The optimal cut-off points for SHR and GG to predict prognostic outcome after MT in LVOS patients were 0.89(sensitivity of 0.635 and specificity of 0.701) and − 0.53(sensitivity of 0.573 and specificity of 0.749) respectively. Moreover, the area under the curve (AUC) for the ability of SHR and GG levels to predict poor outcomes was 0.691 and 0.682, respectively (Fig. 4).

### Relationship between SHR, GG and HT

All of the patients were divided into two groups according to HT, of which 111(26.2%) patients experienced HT. In the univariate analysis, we found that antiplatelet/anticoagulant history (*P* = 0.040), IT (*P* = 0.003), OTP (*P* = 0.001), OTR (*P* < 0.001), SHR > 0.89 (*P* < 0.001) and GG>-0.53 (*P* = 0.001) differed between the two groups (Online Supplemental Table S1). We included variables with *P* < 0.05 in a multifactorial analysis, wherein we initially included clinical data (admission NIHSS, admission ASPECT and SHR > 0.89/GG>-0.53) and subsequently included surgical data (OTR; due to the fact the time period of OTR contains OTP, we only included OTR); in addition, pharmacological treatment was included (IT, and antiplatelet/anticoagulant history). After adjusting for the inclusion of different variables, all of the results showed that SHR > 0.89 and GG>-0.53 increased the risk of HT. In addition, IT, antiplatelet history and low ASPECT increased the risk of HT (**Online Supplemental Tables S2 and S3**).

## Discussion

A number of studies have mentioned that SIH impairs the prognosis of MT patients; however, only a few studies have reasonably quantified this effect. We evaluated SHR and GG to assess the occurrence of SIH, and we found that the incidence of SIH was high base on either the occurrence of 45.2% (SHR) or 40.2% (GG), which was much higher than the proportion of patients with diabetes and also higher than the proportion of patients with SIH as defined by previous studies. In our study, we found that high SHR and high GG independently predicted the outcome after MT. Second, SHR and GG increased the risk of HT in patients who underwent MT.

There have been a number of studies on SIH, but the definition of SIH has been shown to vary. In recent years, some studies have used an increase in absolute blood glucose (fasting blood glucose or random blood glucose levels) to indicate the occurrence of SIH[9, 16]. The increase in absolute blood glucose does suggest that stress is occurring in some patients, but it also has the obvious drawback of failing to take into consideration the effects of background blood glucose, which corresponding affects the final outcome. Similarly, the SHINE study demonstrated that positive interventions based on the blood glucose range did not improve the prognosis, but rather increased the incidence of hypoglycaaemic events [17]. Thus, absolute glucose elevation alone is not a good response to stressful events, and these glucose interventions may need to be considered with respect to the background glucose conditions. In our study, both SHR and GG referenced both background glucose (ADAG) and absolute glucose (FBG) levels, which may be a relatively reasonable choice, however, this may require a multicentre prospective study to confirm. Although some studies have also used SHR (FBG/HbA1c, RBG/HbA1c), these studies were mostly conducted in nondiabetic patients [18, 19], and it is clearly unadvisable to ignore the occurrence of SIH in diabetic patients. A single-centre, small sample study by Yang et al. [11] showed that the SHR and GG can be used to assess the prognosis of acute stroke patients with diabetes. Additionally, a study based on the Chinese Stroke Center Alliance (CSCA) database showed similar findings, with the SHR being a prognostic indicator for diabetic patients with AIS and associated with the risk of in-hospital death[20]. Our study was conducted in a total population and the prevalence of SIH in diabetic patients was over 50%, regardless of whether it was assessed by SHR > 0.89 or GG > -0.53 (specific data not provided in the text). Again, our study showed that both the SHR and GG were reliable indicators for assessing the outcomes of all MT patients. Several studies have supported our viewpoint; for example, Yuan et al. [21] showed that a higher SHR increased the risk of haemorrhagic transformation in patients with AIS regardless of whether or not they had diabetes. A recent study has also shown that the SHR is an important predictor of outcome for patients receivingIVT[22].Also Chen et al. showed that the SHR was associated with the prognosis of MT[23].

The detailed mechanism of the SHR and prognosis of MT patients is not clear, however, some viewpoints have been accepted by most scholars. SIH involves an elevation of blood glucose secondary to major illnesses such as trauma, stroke or even surgery[5]. SIH is partially a manifestation of the stress response. Therefore, it leads to activation of the sympathetic and hypothalamic pituitary-adrenal axis[5]. The release of factors such as cortisol and catecholamines during this process leads to an increase in blood glucose, which is mainly accomplished via gluconeogenesis[5, 6]. Incidentally, glucose is a proinflammatory mediator in the body[6]. SIH causes an increase in reactive oxygen species in the body,thus leading to oxidative stress and an inflammatory cascade that disrupts the immune system[21]; thus pathway, ultimately lead to the development of various infection-related complications[24, 25]. It has also been suggested that a higher SHR leads to an increased incidence of stroke-associated pneumonia and a poor outcome[25]. SIH can leads to the destruction of the vascular endothelium and a decrease in nitric oxide release, thus limiting arterial dilatory reserve and affecting reperfusion[25, 26]. Moreover, SIH affects the action of clotting factors, whereas hyperglycaemic states induce platelet aggregation, which together lead to a prothrombotic state[5, 27]. Furthermore, it has been widely accepted that SIH increases the incidence of cerebral edema and haemorrhagic transformation[4, 28].

IR is a common phenomenon of elevated blood glucose in stroke patients[7]. Insulin regulates blood glucose by promoting glucose uptake by skeletal muscle, cardiac muscle and other adipose tissues, as well as by inhibiting hepatic glycogenolysis and gluconeogenesis. When these effects are reduced, insulin resistance occurs, which results in an increase in blood glucose[7, 29]. The inflammatory state in patients with stroke and cardiovascular disease reduces insulin sensitivity[29]. Further pro-inflammatory factors exacerbate ischaemic injury in insulin-resistant patients[30].

It is noteworthy that complete recanalization was a factor that improved prognosis in previous studies; however, mTICI had no statistical significance in our multifactorial analysis, but was significant in the univariate analysis. We may be able to explain this conclusion from a statistical point of view. The reperfusion success rate of the 5 major randomized trials was approximately 70%[1], whereas the recently published RESCUE BT study was as high as 90% [31], which may be attributed to the maturation and refinement of MT-related techniques. In contrast, in our study, the reperfusion success rate was 89.6% (favourable outcome,93.5% vs. poor outcome, 84.9%; *P* = 0.004). Moreover, the small sample size of reperfusion failure resulted in its nonsignificance in the multiple regression analysis.

It is worth noting that the present study had some limitations. First, our study was a single-centre retrospective study that requires a larger sample size and prospective studies for support. Second, due to the small sample size of diabetic patients in this study, we failed to analyse in different background glucose groups. In addition, although we did not perform glycaemic interventions prior to FBG collection whenever possible, we intervened in a very small number of patients by using subcutaneous insulin injection in consideration of their condition, however we did not exclude these patients from the study. Finally, we were unable to collect blood glucose related metrics over multiple time periods, and a subcutaneous implantable blood glucose monitoring device may be a good option.

## Conclusion

This study showed that both SIH markers(SHR and GG) were significantly associated with poor outcomes in MT patients and increased risk of HT.

## Electronic supplementary material

Below is the link to the electronic supplementary material.

Supplementary Material 1

Supplementary Material 2

Supplementary Material 3

## Data Availability

The data are available from corresponding author(Xianjun Huang, Email: doctorhuangxj@hotmail.com) upon reasonable request.

## Abbreviations

MT: Mechanical thrombectomy
SIH: Stress-induced hyperglycemia
HbA1c: Glycated hemoglobin
SHR: Stress hyperglycemia ratio
GG: Glycemic gap
mRS: Modified Rankin Scale score
ADAG: A1c-Derived Average Glucose
NIHSS: National Institutes of Health Stroke Scale
ASPECT: Alberta Stroke Program Early CT
TOAST: Trial of Org 10172 in Acute Stroke Treatment
LAA: Large-artery atherosclerosis
ICA: Internal carotid artery
MCA(M1/M2) M1/M2: Middle cerebral artery segment
OTP: Onset-to-puncture time
OTR: Onset-to-reperfusion time
mTICI: Modified Thrombolysis in Cerebral Infarction
