# Supplementary material for: New stress-induced hyperglycaemia markers predict prognosis in patients after mechanical thrombectomy
Source: BMC Neurol. 2023 Mar 30;23:132. doi: 10.1186/s12883-023-03175-w (PMC10061963; doi:10.1186/s12883-023-03175-w)
Supplement: Supplementary file 1 — Supplementary Material 1 [file 12883_2023_3175_MOESM1_ESM.docx]

S1 Baseline clinical characteristics of patients with No-HT and HT groups

|  | No-HT  (n=312) | HT  (n=111) | *P*-value |
| --- | --- | --- | --- |
| Age[year，*M* (*Q_1_*, *Q_3_)*] | 70(62,77) | 72(65,77) | 0.221 |
| Male [n, (%)] | 190(60.9) | 60(54.1) | 0.208 |
| Medical history [n, (%)] |  |  |  |
| Hypertension | 198(63.5) | 71(64.0) | 0.925 |
| Diabetes mellitus | 44(14.1) | 15(13.5) | 0.878 |
| Atrial fibrillation | 158(42.0) | 124(64.6) | 0.268 |
| Antiplatelets/anticoagulants history [n, (%)] |  |  | 0.040 |
| No | 259(83.0) | 81(73.0) |  |
| Antiplatelets | 35(11. 2) | 23(20.7) |  |
| Anticoagulants | 18(5.8) | 7(6.3) |  |
| IT [n, (%)] | 29(9.3) | 22(19.8) | 0.003 |
| Baseline SBP^a^[mmHg，*M* (*Q_1_*, *Q_3_)*] | 153(140,168) | 150(137,168) | 0.179 |
| Baseline DBP^b^[mmHg，*M* (*Q_1_*, *Q_3_)*] | 83(74,93) | 83(74,91) | 0.649 |
| Admission NIHSS [*M* (*Q_1_*, *Q_3_)*] | 11(13,17) | 14(12,18) | 0.021 |
| Admission ASPECT [*M* (*Q_1_*, *Q_3_)*] | 9(8,10) | 8(6,9) | <0.001 |
| TOAST classification [n, (%)] |  |  | 0.083 |
| LAA | 96(30.8) | 23(20.7) |  |
| Cardioembolic | 180(57.7) | 77(69.4) |  |
| Others | 36(11.5) | 11(9.9) |  |
| Occlusion location [n, (%)] |  |  | 0.114 |
| ICA | 112(35.9) | 52(46.8) |  |
| MCA(M1) | 168(53.8) | 48(43.2) |  |
| MCA(M2) | 32(10.3) | 11(9.9) |  |
| OTP [min, *M* (*Q_1_*, *Q_3_)*] | 283(217,360) | 330(249,420) | 0.001 |
| OTR^c^ [min, *M* (*Q_1_*, *Q_3_)*] | 348(270,430) | 405(330,510) | <0.001 |
| Collateral score^d^ [n, (%)] |  |  | 0.130 |
| Grade 0 | 32(10.3) | 17(15.3) |  |
| Grade 1 | 70(22.6) | 31(27.9) |  |
| Grade 2 | 208(67.1) | 63(56.8) |  |
| mTICI,2b/3 [n, (%)] | 283(90.7) | 96(86.5) | 0.211 |
| SHR [*M* (*Q_1_*, *Q_3_)*] | .85(.74,.1.00) | .95(.83,1.17) | <0.001 |
| GG [*M* (*Q_1_*, *Q_3_)*] | -.99(-1.75,.00) | -.31 (-1.10,1.05) | <0.001 |
| SHR (>0.89) [n, (%)] | 124(39.7) | 67(60.4) | <0.001 |
| GG (>-0.53) [n, (%)] | 110(35.3) | 59(53.2) | 0.001 |

**Abbreviations:** HT, haemorrhagic transformation; SBP, systolic blood pressure; DBP, diastolic blood pressure; 1mmHg=0.133kPa; IT, Intravenous Thrombolysis; NIHSS, National Institutes of Health Stroke Scale; ASPECT, Alberta Stroke Program Early CT; TOAST, Trial of Org 10172 in Acute Stroke Treatment; LAA, large-artery atherosclerosis; ICA, internal carotid artery; MCA(M1/M2) M1/M2 middle cerebral artery segment, OTP, onset-to-puncture time; OTR, onset-to-reperfusion time; mTICI, modified Thrombolysis in Cerebral Infarction; SHR, stress hyperglycaemia ratio; GG, glycaemic gap.

a:13 patients lost data on SBP

b:13 patients lost data on DBP

c:1 patient lost data on OTR

d:2 patients lost data on Collateral score
