# Supplementary material for: New stress-induced hyperglycaemia markers predict prognosis in patients after mechanical thrombectomy
Source: BMC Neurol. 2023 Mar 30;23:132. doi: 10.1186/s12883-023-03175-w (PMC10061963; doi:10.1186/s12883-023-03175-w)
Supplement: Supplementary file 2 — Supplementary Material 2 [file 12883_2023_3175_MOESM2_ESM.docx]

S2 Multi-factor analysis for HT associated with SHR

|  | Adjusted OR **^a^**  (95%CI) | *P*-value | Adjusted OR **^b^**  (95%CI) | *P*-value | Adjusted OR **^c^**  (95%CI) | *P*-value |
| --- | --- | --- | --- | --- | --- | --- |
| SHR (>0.89) | 1.804(1.124~2.896) | 0.015 | 1.776(1.105~2.855) | 0.018 | 1.764(1.086~2.866) | 0.022 |
| Admission NIHSS | 1.025(0.976~1.076) | 0.330 | 1.030(0.980~1.082) | 0.248 | 1.031(0.979~1.084) | 0.247 |
| Admission ASPECT | 0.862(0.774~0.960) | 0.007 | 0.866(0.777~0.965) | 0.009 | 0.859(0.769~0.960) | 0.007 |
| OTR^*^ | ---- | ---- | 1.000(1.000~1.001) | 0.261 | 1.009(1.003~1.015) | 0.427 |
| IT | ---- | ---- | ---- | ---- | 2.738(1.450~5.167) | 0.002 |
| Antiplatelets/anticoagulants history |  |  |  |  |  | 0.050 |
| Antiplatelets vs No | ---- | ---- | ---- | ---- | 2.114(1.137~3.930) | 0.018 |
| Anticoagulants vs No | ---- | ---- | ---- | ---- | 1.540(0.602~3.943) | 0.368 |

**a**: adjusted for SHR (>0.89), admission NIHSS and admission ASPECT

**b**: adjusted for **a**, and OTR

**c**: adjusted for **b**, IT and Antiplatelets/anticoagulants history

**Abbreviations:** HT, haemorrhagic transformation; NIHSS, National Institutes of Health Stroke Scale; ASPECT, Alberta Stroke Program Early CT; OTR, onset-to-reperfusion time; IT, intravenous thrombolysis; SHR, stress hyperglycaemia ratio.

*:1 patient lost data on OTR
