# Supplementary material for: New stress-induced hyperglycaemia markers predict prognosis in patients after mechanical thrombectomy
Source: BMC Neurol. 2023 Mar 30;23:132. doi: 10.1186/s12883-023-03175-w (PMC10061963; doi:10.1186/s12883-023-03175-w)
Supplement: Supplementary file 3 — Supplementary Material 3 [file 12883_2023_3175_MOESM3_ESM.docx]

S3 Multi-factor analysis for HT associated with GG

|  | Adjusted OR **^a^**  (95%CI) | *P*-value | Adjusted OR **^b^**  (95%CI) | *P*-value | Adjusted OR **^c^**  (95%CI) | *P*-value |
| --- | --- | --- | --- | --- | --- | --- |
| GG (>-0.53) | 1.698(1.065~2.709) | 0.026 | 1.694(1.061~2.705) | 0.027 | 1.699(1.050~2.749) | 0.031 |
| Admission NIHSS | 1.028(0.978~1.079) | 0.277 | 1.033(0.983~1.086) | 0.199 | 1.033(0.982~1.087) | 0.205 |
| Admission ASPECT | 0.858(0.770~0.955) | 0.005 | 0.863 (0.775~0.962) | 0.008 | 0.856(0.767~0.957) | 0.006 |
| OTR^*^ | ---- | ---- | 1.000(1.000~1. 001) | 0.217 | 1.000(1.000~1.001) | 0.364 |
| IT | ---- | ---- | ---- | ---- | 2.785(1.475~5.259) | 0.002 |
| Antiplatelets/anticoagulants history |  |  |  |  |  | 0.054 |
| Antiplatelets vs No | ---- | ---- | ---- | ---- | 2.085(1.122~3.876) | 0.020 |
| Anticoagulants vs No | ---- | ---- | ---- | ---- | 1.554(0.606~3.983) | 0.358 |

**a**: adjusted for GG (>-0.53), admission NIHSS and admission ASPECT

**b**: adjusted for **a**, and OTR

**c**: adjusted for **b**, IT, Antiplatelets/anticoagulants history

**Abbreviations:** HT, haemorrhagic transformation; NIHSS, National Institutes of Health Stroke Scale; ASPECT, Alberta Stroke Program Early CT; OTR, onset-to-reperfusion time; IT, intravenous thrombolysis; GG, glycaemic gap.

*:1 patient lost data on OTR
